# HPV16 E5 and KGFR/FGFR2b interplay in differentiating epithelial cells

**DOI:** 10.18632/oncotarget.803

**Published:** 2013-02-22

**Authors:** Valeria Purpura, Francesca Belleudi, Silvia Caputo, Maria Rosaria Torrisi

**Affiliations:** ^1^ Istituto Pasteur-Fondazione Cenci Bolognetti, Dipartimento di Medicina Clinica e Molecolare, Sapienza Università di Roma, Italy; ^2^ Azienda Ospedaliera S. Andrea, Rome, Italy

**Keywords:** HPV16 E5, FGFR2b, KGFR, differentiation, human keratinocytes

## Abstract

The E5 oncogenic protein of the human papillomavirus type 16 (HPV16 E5) cooperates in epithelial transformation perturbing the behaviour of differentiating suprabasal cells. Among the receptor tyrosine kinases deregulated by 16E5 expression, the key paracrine mediator of epithelial homeostasis keratinocyte growth factor receptor (KGFR/FGFR2b) is altered in its signaling and endocytic traffic in undifferentiated keratinocytes expressing 16E5 and it would represent a major target of the viral protein in differentiated cells. With the aim to specifically address the possible interplay of 16E5 with KGFR/FGFR2b in cells already committed to differentiation, we took advantage of an in vitro model for forced overexpression or depletion of KGFR in E5 expressing human keratinocytes under synchronous waves of differentiation. Quantitative RT-PCR, biochemical and immunofluorescence analysis showed that KGFR down-modulation is responsible for a E5-mediated decrease of the early differentiation marker K1 and that the receptor re-expression as well as triggering of its kinase activity and signaling are able to efficiently counteract the impairment of differentiation, providing a further demonstration of the tumor-suppressive role of KGFR in the new unexplored context of HPV16 E5-mediated carcinogenesis. In addition, KGFR induced a ligand-dependent decrease of p63 through a miR-203 independent mechanism and this effect was blocked by inhibition of the PI3K/Akt signaling, which is the main pathway involved in KGFR-dependent keratinocyte differentiation, suggesting that alterations of the KGFR/p63 crosstalk are responsible for the impairment of keratinocyte differentiation induced by 16E5 and that the opposite tumor-suppressive action of KGFR and oncogenic role of E5 might both involve p63.

## INTRODUCTION

The E5 oncogenic protein of the human papillomavirus type 16 (HPV16 E5) is known to be involved in epithelial transformation and cervical carcinogenesis through cooperation with the other two viral oncogenes E6 and E7 [1, 2]. Although the molecular mechanisms of the E5 oncogenic activities are still poorly defined, this protein would play its major roles at the level of differentiating cells by perturbing their proliferation and differentiation [3, 4]. Consistent with this hypothesis, expression of the protein might be mostly confined to the suprabasal layer of the epithelial tissues where it may act sustaining cell growth through deregulation of receptor tyrosine kinases (RTKs) signaling [1].

In agreement with the possibility of the existence of a functional crosstalk among 16E5 and RTKs in differentiating epithelia, we have recently reported 5 that 16E5 expression induces down-modulation of the keratinocyte growth factor receptor (KGFR/FGFR2b), a splicing transcript variant of the fibroblast growth factor receptor 2 (FGFR2), which plays a key role in the balance between epithelial growth and differentiation 6. KGFR is mostly distributed on suprabasal cells [7, 8] and its expression is up-regulated during keratinocyte differentiation [9, 10]. Moreover, differently from most RTKs, KGFR appears to play an unusual and unique role in epithelial cells, acting as a tumor suppressor *in vitro* and *in vivo* [6, 11, 12]. Based on these findings, we have proposed that the inverse correlation in the expression of 16E5 and KGFR would lead to opposite and interplaying roles in epithelial homeostasis and tumorigenesis. Accordingly with our working hypothesis, the skin KGFR/FGFR2b-deficient mouse phenotype [13, 14] closely reminds the transgenic mouse for epithelial targeted 16E5 expression [15], since both models are characterized by epidermal hyperplasia and impairment of differentiation as well as by a similar behaviour in chemical-induced carcinogenesis.

Therefore, with the aim to specifically address the possible interplay of 16E5 with KGFR/FGFR2b in cells already committed to differentiation, we took advantage of an *in vitro* model, recently developed in our laboratory [10], to modulate receptor expression in human cultured keratinocytes under synchronous waves of differentiation induced by treatment with Thapsigargin (TG), an inhibitor of Ca-ATPase pump family [16]. Using this strategy of forced KGFR overexpression or depletion under controlled triggering of cell differentiation, we were able to demonstrate that KGFR is a crucial player in the induction of keratinocyte early differentiation and that the PI3K/Akt signaling pathway is involved in such receptor-mediated function 10. In the present study, using this approach we focused on the HPV16 E5 ability to regulate KGFR expression and signaling in differentiating cells and we investigated the possible counteracting effect exerted by receptor activation.

## RESULTS

### KGFR and K1 are down-modulated by HPV 16E5 in differentiating keratinocytes

**Figure 1:**
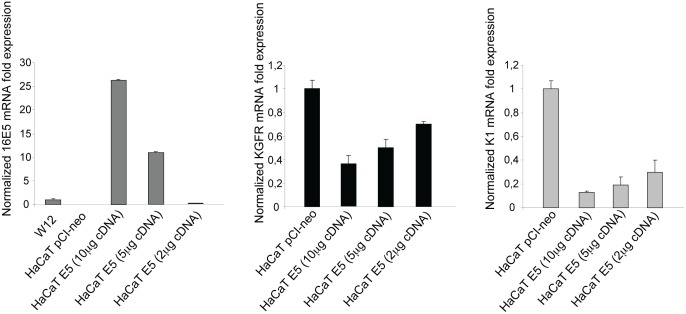
16E5 induces a dose-dependent down-regulation of KGFR and K1 mRNA transcripts HaCaT cells were transiently transfected using decreasing amounts of pCI-neo E5-HA expression vector (10μg, 5μg and 2μg) (HaCaT E5) or using the empty vector alone (HaCaT pCI-neo). After transfection, the 16E5 mRNA (left panel), KGFR mRNA (central panel) and the early differentiation marker K1 mRNA (right panel) transcript levels were quantitated by real-time relative RT-PCR. The decreasing 16E5 mRNA expression levels were normalized with respect to the levels of the viral protein mRNA in the subclone W12p6 of the HPV16-positive cervical epithelial cell line W12 (left panel). Both the receptor and K1 mRNA progressively increases in cells expressing decreasing doses of 16E5.

We have recently demonstrated a key role of KGFR expression and signaling in the induction of human keratinocyte early differentiation [10]. Since we have also shown that KGFR is down-modulated by the expression of HPV 16E5 at both transcript and protein levels [6], here we investigated the possible contribution of KGFR down-modulation to the inhibition of keratinocyte early differentiation induced by the expression of the viral protein. Therefore, with the aim to analyze the interplay between the two 16E5-mediated events, we used the human keratinocyte HaCaT cell line, spontaneously immortalized from a primary culture of keratinocytes and widely used as a model of keratinocyte differentiation and stratification [9, 17]. Pre-confluent cells were transiently transfected with pCI-neo E5-HA expression vector [21] (HaCaT E5) or with the empty vector alone (HaCaT pCI-neo) as previously described [5]. Decreasing amounts of 16E5 cDNA were used to assess the dose-dependency of the effects. The mRNA transcript levels of 16E5 and KGFR as well as of the early differentiation marker keratin 1 (K1) were quantitated by real-time relative RT–PCR using β-actin as housekeeping gene. The decreasing 16E5 mRNA expression levels were normalized with respect to the levels of the viral protein mRNA in the subclone W12p6 of the HPV16-positive cervical epithelial cell line W12 [18]. The results showed that, as expected 5, the expression of 16E5 led to a clear decrease of KGFR expression (Fig. 1, central panel). The specificity of such down-modulation was confirmed by the progressive increase of the receptor mRNA in cells expressing decreasing doses of 16E5 (Fig. 1). In addition, the expression of 16E5 induced a decrease of K1 mRNA expression and this effect also appeared dose-dependent (Fig. 1, right panel). This finding is in agreement with the decrease of K1 expression observed in the suprabasal layer of organotypic culture of HaCaT cells expressing 16E5 [23]. Thus, 16E5 expression is able to down-regulate both KGFR and K1 transcripts, although the basal expression of these two proteins are quite low in pre-confluent, undifferentiated keratinocytes [10].

**Figure 2:**
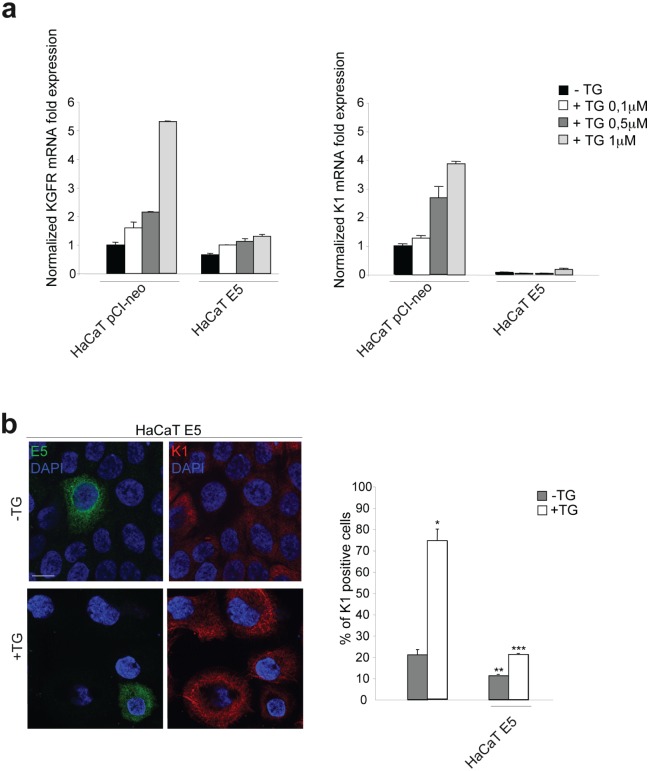
16E5 down-regulates KGFR and K1 at both transcript and protein levels in TG-treated differentiating keratinocytes (a) HaCaT pCI-neo and HaCaT E5 cells were treated with different doses of TG (0.1μM, 0.5μM and 1μM) for 1h at 37°C. Cells treated with equal amount of DMSO solvent were used as a control. KGFR mRNA (left panel) and K1 mRNA (right panel) transcript levels were quantitated by real-time relative RT-PCR: a clear inhibition on KGFR and K1 transcription is found at all TG doses in HaCaT E5 cells compared to HaCaT pCI-neo cells. (b) HaCaT E5 cells were treated with TG 1μM as above, while the control cells were kept in DMSO alone. Double immunofluorescence was performed using anti-HA monoclonal antibody, to visualize 16E5 protein, and anti-K1 polyclonal antibodies. 16E5 staining is localized in cytoplasmic reticular structures, while K1 staining appears cytosolic and filamentous. The K1 signal is decreased in cells expressing 16E5 in both TG-untreated and TG-treated cultures. Cell nuclei were visualized by DAPI. Quantitative immunofluorescence analysis shows that the decrease of the percentage of K1 positive cells induced by 16E5 expression was particularly evident in TG-treated compared to TG-untreated cultures. The quantitative analysis was assessed by counting for each sample a total of 50 cells, randomly observed in 10 microscopic fields from three different experiments. Cut-off of the K1 signal intensity was determined for TG-treated and control samples as described in Materials and Methods. Results are expressed as mean values ± standard errors (SE). Student's t test was performed and significance level has been defined as p<0,05: *p<0,001 vs the corresponding untreated cells; **p<0,01 vs the corresponding surrounding cells that do not show E5 expression; ***p<0,01 vs the corresponding surrounding cells that do not show E5 expression. Bar: 10 μm

To deeply analyze the 16E5 impact on keratinocyte differentiation and to evaluate the role played by KGFR down-modulation in this process, we took advantage of our newly developed *in vitro* model of synchronous receptor modulation and forced cell differentiation [10] through treatment with Thapsigargin (TG), an inhibitor of Ca-ATPase pump family [16]. In fact, using this model, we have recently demonstrated that TG treatment is able to generate an homogenous population of differentiating HaCaT cells expressing increasing amount of KGFR and K1 [10]. Therefore, to analyze the effects of 16E5 in differentiating cells, pre-confluent HaCaT pCI-neo or HaCaT E5 cells were treated with three different doses of TG as reported in Materials and Methods, while control cells were left in an equal amount of the solvent dimethyl sulfoxide (DMSO). Real-time RT-PCR showed a TG dose-dependent progressive increase of KGFR mRNA in pCI-neo cells, which was well evident upon 1μM TG stimulation. In contrast, in cells expressing 16E5 a clear inhibition on KGFR transcription was found at all TG doses (Fig. 2a, left panel).

To analyze whether the down-modulating effect on KGFR expression induced by 16E5 could be accompanied by the inhibition of K1 expression also in keratinocytes forced to differentiate, parallel real-time RT-PCR was performed to quantitate the K1 transcript levels: the inhibition of K1 expression was clearly evident at all doses of TG compared to control pCI-neo cells (Fig. 2a, right panel). Interestingly, the block of K1 expression appeared independent on TG treatment (Fig. 2a, right panel). Double immnunofluorescence analysis, performed in HaCaT E5 cells using anti-HA monoclonal antibody to visualize 16E5 protein and anti-K1 polyclonal antibodies showed that the viral protein signal was localized in cytoplasmic reticular structures (Fig. 2b), probably corresponding to the endoplasmic reticulum [5, 24], while K1 staining appeared cytosolic and filamentous (Fig. 2b). The K1 signal was decreased in 16E5 expressing cells in both TG-untreated undifferentiated cultures and in cells treated with 1μM TG to induce differentiation. The immunofluorescence quantitative analysis of the percentage of K1 positive cells revealed that the 16E5-mediated inhibition of the early differentiation was particularly evident upon TG treatment (Fig. 2b). Thus, the role of 16E5 in the HPV16-induced impairment of the early differentiation program is mostly exerted at the level of cells committed to differentiation.

### KGFR down-modulation is responsible for 16E5-mediated decrease of early differentiation

**Figure 3:**
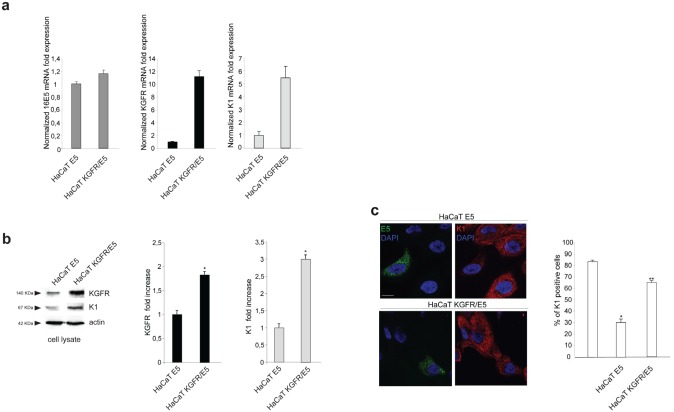
KGFR expression counteracts the 16E5-mediated down-modulation of K1 (a) HaCaT cells were transiently transfected with 16E5 (HaCaT E5) or cotransfected with 16E5 and KGFR (HaCaT KGFR/E5) and then treated with TG as above. the 16E5 mRNA (left panel), KGFR mRNA (central panel) and K1 mRNA transcript levels (right panel) were quantitated by real-time relative RT-PCR: a clear fold increase in both KGFR mRNA (central panels) and K1 mRNA (right panels) expression is observed in HaCaT KGFR/E5 cells compared to HaCaT E5 cells. (b) Western blot analysis was performed in HaCaT E5 and HaCaT KGFR/E5 cells treated with TG as above using anti-K1 polyclonal antibodies and with anti-Bek polyclonal antibodies, which recognize the KGFR/FGFR2b protein. The KGFR band is more visible and K1 band is increased upon KGFR transfection. The equal loading was assessed with anti-actin antibody. For densitometric analysis of the band corresponding to K1 and KGFR proteins the values from three independent experiments were normalized, expressed as fold increase and reported in graph as mean values ± standard deviation (SD). Student't test was performed and significance levels have been defined as above: *p<0,05 vs the corresponding HaCaT E5 cells. (c) Quantitative immunofluorescence analysis performed using anti-HA and anti-K1 antibodies as above shows a recovery in the percentage of K1 positive cells, decreased in HaCaT E5 cells, when KGFR is co-expressed with the viral protein. The quantitative analysis was assessed as above. Results are expressed as mean values ± standard errors (SE). Student's t test was performed and significance level has been defined as above *p<0,0001 vs the corresponding surrounding cells that do not show E5 expression; **p<0,001 vs the corresponding HaCaT E5 cells. Bar: 10 μm

To evaluate if the effect of 16E5 on K1 expression could be mediated by its ability to induce KGFR down-modulation, we induced the rapid, forced and synchronous modulation of the receptor expression by transient transfection of KGFR cDNA or by microinjection of KGFR siRNA in 16E5-expressing cells. To first analyze the effect of KGFR overexpression, HaCaT cells were singly tranfected with 16E5 (HaCaT E5) or cotransfected with 16E5 and KGFR (HaCaT KGFR/E5) and then treated with TG as above to induce differentiation. The transcript levels of 16E5, KGFR and K1 were analyzed by real-time RT-PCR. The results showed that, in KGFR/E5 cells, K1 mRNA levels were 5 fold increased if compared to those detected in E5 cells (Fig. 3a, right panel). The enhanced expression of K1 induced by KGFR overexpression was also validated at the protein level by Western blot analysis using anti-K1 polyclonal antibodies; the band at the molecular weight corresponding to K1 protein appeared increased in HaCaT KGFR/E5 cells if compared to that observed in HaCaT E5 cells (Fig. 3b). Immunoblot analysis using anti-Bek polyclonal antibodies, which recognize the intracellular portion of the two splicing variants KGFR/FGFR2b and FGFR2c, showed that the 140 KDa specific band corresponding to the receptor molecular weight was clearly enhanced upon KGFR transfection (Fig. 3b). Quantitative immunofluorescence analysis, performed using anti-HA and anti-K1 antibodies as above, revealed that the decrease in the percentage of K1 positive cells in HaCaT E5 cells (Fig. 3c) appeared significantly recovered when KGFR was co-expressed with the viral protein (Fig. 3c). These results strongly indicated that, in presence of 16E5, the forced overexpression of KGFR is sufficient to counteract the down-modulating effect exerted by the viral protein on K1 gene expression.

**Figure 4:**
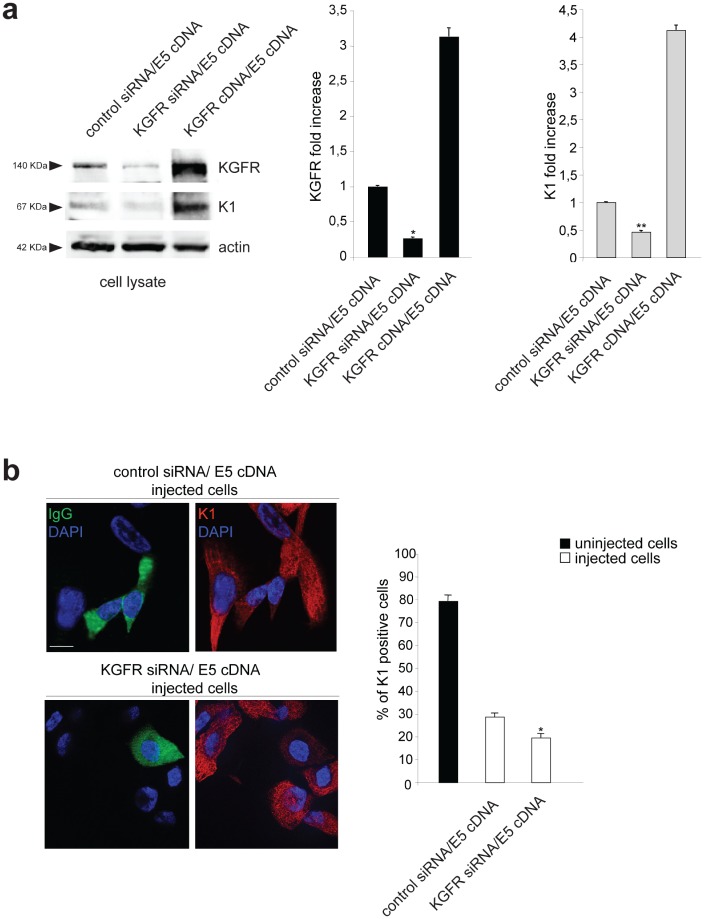
KGFR silencing enhances the effect of 16E5 on K1 expression (a) HaCaT cells were cotransfected with small interfering RNA for FGFR2/Bek (KGFR siRNA) to obtain receptor silencing and with 16E5 cDNA to obtain the viral protein expression and then treated with TG as above. Cotransfection of 16E5 cDNA with KGFR cDNA or with an unrelated siRNA were used as controls. Western blot analysis using anti-Bek and anti-K1 antibodies shows an evident decrease of both endogenous KGFR and K1 marker in HaCaT cells cotransfected with KGFR siRNA and 16E5 compared to control siRNA/16E5 cDNA cells. An increase of K1 protein levels is evident in KGFR cDNA/E5 cDNA cells. The equal loading was assessed with anti-actin antibody. The densitometric analysis and Student't test were performed and significance levels have been defined as above: *p<0,005 vs the corresponding control siRNA/E5 cDNA transfected cells; **p<0,05 vs the corresponding control siRNA/E5 cDNA transfected cells. (b) HaCaT cells were coinjected with KGFR siRNA, 16E5 cDNA and mouse IgG to identify the injected cells. Coinjection of 16E5 cDNA and an unrelated siRNA was performed as a control. After injection, cells were treated with TG as above. Quantitative immunofluorescence analysis using anti-K1 antibodies shows that the percentage of K1 positive cells, which is strongly decreased by 16E5 expression in HaCaT control siRNA/ E5 cDNA if compared to the surrounding uninjected cells, is further diminuished upon KGFR depletion in KGFR siRNA/E5 cDNA coinjected cells. The quantitative analysis was assessed as previously described. Results are expressed as mean values ± standard errors (SE). Student's t test was performed and significance level has been defined as above: *p<0,005 vs the corresponding control siRNA/E5 cDNA injected cells. Bar: 10 μm

To demonstrate that the specific outcome of the 16E5 presence on cell differentiation might be a consequence of the viral protein-induced down-modulation of KGFR, we analyzed the effect of the receptor depletion on K1 expression cotransfecting HaCaT cells with small interfering RNA for FGFR2/Bek (KGFR siRNA) to obtain receptor silencing and with E5 cDNA to obtain the viral protein expression. Cotranfection of E5 cDNA with KGFR cDNA or with an unrelated siRNA were used as controls. After transfection, cells were treated with TG as above. Western blot analysis showed that, while K1 appeared increased when KGFR was overexpressed, the receptor depletion induced an evident decrease of both KGFR and K1 compared to control cells (Fig. 4a). Thus, in the presence of 16E5, the forced modulation of KGFR induces a corresponding modulation of K1 expression.

These results were validated by immunofluorescence analysis co-injecting cells with KGFR siRNA, E5 cDNA and mouse IgG to identify the injected cells. Coinjection of E5 cDNA and an unrelated siRNA was performed as control. After injection, cells were treated with TG as above. Quantitative immunofluorescence analysis showed that the percentage of K1 positive cells, which in control cells appeared strongly decreased for the injected cells compared to the surrounding uninjected (Fig. 4b), was further diminished by KGFR depletion in KGFR siRNA/E5 cDNA coinjected cells (Fig. 4b). These results indicate that KGFR silencing enhances the down-modulating effect exerted by 16E5 protein on K1 expression.

### KGFR kinase activity and signaling are required to counteract the 16E5-induced impairment of differentiation

**Figure 5:**
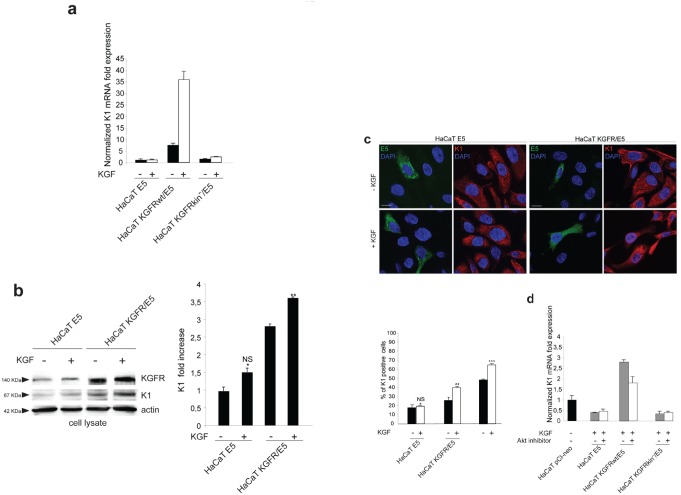
The KGFR counteracting effect on 16E5 requires receptor activation and PI3K/Akt signaling (a) HaCaT cells transfected with 16E5 (HaCaT E5) or cotransfected with 16E5 and KGFR (HaCaT KGFRwt/E5) or KGFRY656F/Y657F kinase negative mutant (HaCaT KGFRkin^−^/E5) were treated with TG as above, serum starved and then stimulated with 20 ng/ml KGF for 24h at 37°C. The K1 mRNA transcript levels were quantitated by real-time relative RT-PCR: a very strong ligand-dependent increase of K1 mRNA expression is observed in HaCaT KGFRwt/E5 cells, although a clear enhancement of K1 levels is detectable also in absence of ligand stimulation, due to a not complete receptor signaling shut down by serum starvation. No significant changes are found in response to KGF treatment in HaCaT E5 cells, as well as in HaCaT KGFRkin^−^/E5 cells. (b) Western blot analysis on HaCaT E5 and HaCaT KGFR/E5 cells treated with TG and stimulated with KGF as above shows that the band of K1 protein, already increased in HaCaT KGFR/E5 compared to HaCaT E5 cells, is further enhanced in cells stimulated by the ligand. The equal loading was assessed with anti-actin antibody. The densitometric analysis and Student't test were performed and significance levels have been defined as above: *NS vs the corresponding unstimulated HaCaT E5 cells; **p<0,05 vs the corresponding unstimulated HaCaT KGFR/E5 cells. (c) Quantitative immunofluorescence analysis shows a significant ligand-dependent increase of K1 positive cells in KGFR/E5 cells as well as in the surrounding cells that do not express detectable levels of 16E5 protein compared to HaCaT E5 cells. No significant changes are observed in HaCaT E5 cells in response to ligand stimulation. The quantitative analysis was assessed as previously described. Results are expressed as mean values ± standard errors (SE). Student's t test was performed and significance level has been defined as above: *NS vs the corresponding unstimulated HaCaT E5 cells; **p<0,05 vs the corresponding unstimulated HaCaT KGFR/E5 cells; p< 0,001 vs the corresponding unstimulated cells. Bar: 10 μm. (d) HaCaT pCI-neo, HaCaT E5, HaCaT KGFRwt/E5 and HaCaT KGFRkin^−^/E5 cells were serum starved, treated with TG and then stimulated with KGF in presence or not of the Akt inhibitor. Quantitative real-time RT-PCR shows that the inhibition of Akt reduces the counteracting effect exerted by KGFRwt on K1 down-modulation mediated by 16E5 in HaCaT KGFRwt/ E5 cells while it does not affect K1 levels in HaCaT E5 as well as in HaCaT KGFRkin^−^/E5 cells.

Since we have previously demonstrated that the differentiative role of KGFR implies receptor activation and signaling [10], we wondered whether the ligand-dependent activation of KGFR would be required for its counteracting effect on 16E5-induced impairment of early differentiation. To address this point, HaCaT E5 and HaCaT KGFR/E5 transfected cells were triggered to differentiate by TG treatment as above, serum starved and then stimulated with 20 ng/ml KGF for 24 h at 37°C. Cells were alternatively cotransfected with 16E5 and the Y656F/Y657F KGFR kinase negative mutant (KGFR kin^−^) [22]. Quantitative real-time RT-PCR showed a very strong ligand-dependent increase in K1 mRNA expression in KGFR/E5 (Fig. 5a), whereas no significant changes in response to KGF treatment were observed in E5 cells, expressing very low levels of the endogenous receptors due to the viral protein-mediated down-modulation (Fig. 5a). In KGFR/E5 transfected cells, a clear increase of K1 expression was evident also in absence of ligand stimulation as a result of a not complete shut down of the receptor-mediated signaling upon serum starvation (Fig. 5a). This possible explanation was confirmed by the results obtained in cells cotransfected with the kinase negative mutant KGFR, in which the receptor-specific signaling was abolished: in fact, in these cells the K1 mRNA levels appeared comparable to those observed in E5 cells and were not affected by KGF stimulation (Fig. 5a).

Western blot analysis showed that the band corresponding to K1 protein appeared increased in KGFR/E5 compared to E5 cells and clearly enhanced by ligand stimulation (Fig. 5b). Again, the increase of K1 in KGFR cotransfected cells, also when unstimulated, could be ascribed to a partial persistence of receptor signaling upon starvation (Fig. 5b). Parallel quantitative immunofluorescence analysis indicated that, compared to E5 cells, a significant ligand-dependent increase of K1 positive cells was observed in KGFR/E5 cells as well as in the surrounding cells that did not express detectable levels of 16E5 protein, but in which the endogenous KGFRs were up-modulated upon TG stimulation (Fig. 5c). In contrast, no significant ligand-dependent increase of K1 positive-cells was detectable in E5 cells, consistent with the KGFR down-regulation mediated by the viral protein (Fig. 5b).

Since we have previously demonstrated that KGFR expression induces early differentiation through the PI3K/Akt signaling pathway [10], we wondered if this pathway could be responsible also for the counteracting effect exerted by KGFR on 16E5-mediated decrease of K1 expression. To answer this point, HaCaT pCI-neo, HaCaT E5 and HaCaT KGFR/E5 cells were serum starved, treated with TG and then stimulated with KGF in presence or not of Akt inhibitor as reported in Materials and Methods. Alternatively, cells were cotransfected as above with 16E5 and the kinase negative mutant KGFR, (KGFR kin^−^) as negative control. Quantitative real-time RT-PCR clearly showed that the Akt inhibitor did not affect the down-modulation of K1 transcripts induced by 16E5 expression in E5 as well as in KGFRkin^−^/E5 cells (Fig. 5d). In contrast, this inhibitor clearly reduced the counteracting effect exerted by KGFR on the 16E5 action in KGFR/E5 cells (Fig. 5d). These results strongly suggest that the shutting down of KGFR-mediated PI3K/Akt signaling, consequent to receptor down-modulation, is a crucial step for 16E5-mediated impairment of early differentiation.

### KGFR counteracts the up-modulating effect of 16E5 on p63 expression

**Figure 6:**
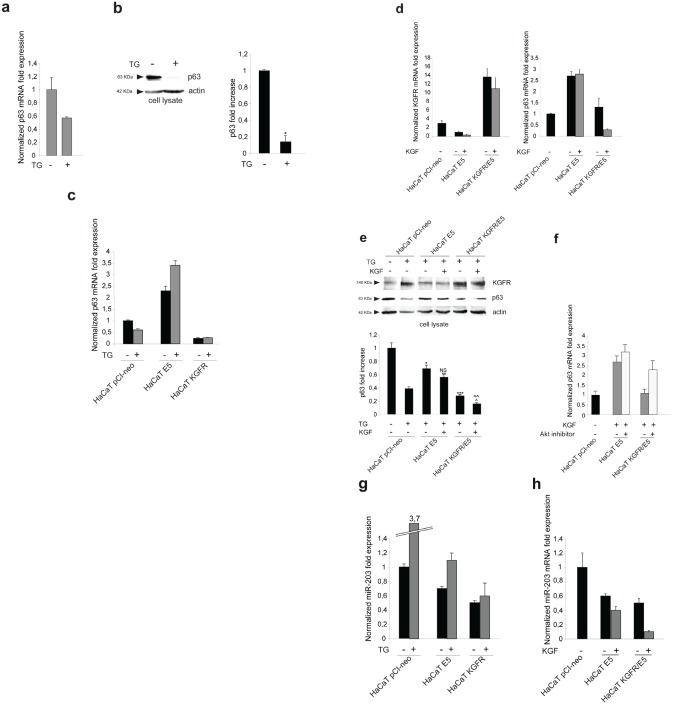
KGFR expression triggers a miR-203-independent down-modulation of p63 and counteracts the p63 up-modulation consequent to 16E5 expression (a) HaCaT cells were treated or not with TG as above and the p63 transcript levels were quantitated by real-time relative RT-PCR: a decrease in p63 mRNA is evident in TG-treated differentiating cells. (b) Western blot analysis was performed in HaCaT cells treated as above using anti-p63 polyclonal antibodies (4A4). The band at the molecular weight corresponding to p63 protein is reduced in cells induced to differentiate compared to unstimulated cells. The equal loading was assessed with anti-actin antibody. The densitometric analysis was performed as above. Student't test was performed and significance levels have been defined as above: *p<0,05. (c) HaCaT cells were alternatively singly transfected with the empty vector pCI-neo, 16E5 or with KGFR and then treated with TG or left in DMSO alone as above. p63 transcript levels were quantitated by real-time RT-PCR: 16E5 up-regulates p63 expression more efficiently in TG-treated than untreated cells while the receptor overexpression induces a decrease of p63 independently from TG stimulus. (d) HaCaT pCI-neo, HaCaT E5 and HaCaT KGFR/E5 cells were treated with TG and then stimulated with KGF as above. Quantitative real-time relative RT-PCR shows that the decrease of p63 mRNA in KGFR/E5 cells is ligand-dependent while the up-modulation of p63 transcript levels in HaCaT E5 cells is not affected by KGF stimulation; the decrease of p63 expression in KGFR/E5 cells compared to cells expressing 16E5 alone is due to a partial persistence of the receptor signaling. (e) Western blot analysis was performed in HaCaT pCI-neo, HaCaT E5 and HaCaT KGFR/E5 cells treated as above. The band corresponding to p63 is decreased in a ligand-dependent manner in HaCaT KGFR/E5, but not in HaCaT E5 cells. The equal loading was assessed with anti-actin antibody. The densitometric analysis and Student't test were performed and significance levels have been defined as above: *p<0,01 vs the corresponding TG-treated HaCaT pCI-neo cells; **NS vs the corresponding KGF-unstimulated HaCaT E5 cells; ***p<0,01 vs the corresponding TG-treated HaCaT pCI-neo cells; ^p<0,005 vs the corresponding TG-treated HaCaT pCI-neo cells; ^^p<0,01 vs the corresponding KGF-stimulated HaCaT KGFR/E5 cells. (f) HaCaT E5 and HaCaT KGFR/E5 cells were treated with TG and then stimulated with KGF in presence or not of the Akt inhibitor as reported in Materials and Methods. Quantitative real-time relative RT-PCR shows that Akt inhibition increases the p63 mRNA expression in KGFR/E5 cells, but it does not alters the up-modulated p63 mRNA amounts in HaCaT E5 cells. (g) HaCaT pCI-neo, HaCaT E5 and HaCaT KGFR cells were treated or not with TG as above and miR-203 levels were quantitated by real-time relative RT-PCR: the down-modulation of miR-203 is evident in cells expressing 16E5 upon TG-treatment, while in HaCaT KGFR cells miR-203 does not appear up-modulated. (h) HaCaT pCI-neo, HaCaT E5 and HaCaT KGFR/E5 cells were treated with TG and then stimulated with KGF with 20 ng/ml. Quantitative real time relative RT-PCR shows that KGFR overexpression does not counteract the down-modulating effect mediated by 16E5 on miR-203 expression level.

Since it has been demonstrated that 16E5 is able to affect the expression of numerous host genes [25] and in particular it has been proposed that this viral protein may suppress differentiation through up-regulation of the transcription factor p63 [26], we wondered whether the counteracting effect exerted by KGFR on 16E5-induced impairment of keratinocyte early differentiation could be, at least in part, due to a receptor ability to contrast the up-regulation of p63. Because it is well known that p63 is expressed in basal keratinocytes and down-modulated during differentiation [27], we first assessed if TG treatment would be able to induce p63 down-modulation at both transcript and protein levels. To this aim, HaCaT cells were treated with TG as above and the transcript levels of p63 were analyzed by real-time RT-PCR. The results showed a decrease of p63 mRNA in TG-treated differentiating cells (Fig. 6a), comparable to that previously described in primary keratinocytes induced to differentiate by high calcium stimulation [28]. The down-modulation of p63 expression upon TG treatment was also validated at the protein level by Western blot analysis: the band at the molecular weight corresponding to p63 protein appeared reduced in cells stimulated to differentiate compared to unstimulated cells (Fig. 6b).

Then we investigated whether 16E5 or KGFR expression would affect p63 expression in undifferentiated as well as differentiating keratinocytes. HaCaT cells were alternatively singly transfected with 16E5, KGFR or with the empty vector pCI-neo; after tranfection, cells were treated with TG or left in DMSO alone as above and p63 transcript level was quantitated by real-time RT-PCR. The results revealed that 16E5 up-regulated p63 expression more efficiently in TG-treated than untreated cells (Fig. 6c), reinforcing the hypothesis that the viral protein affect human keratinocyte early differentiation through the modulation of genes such as KGFR and p63 prevalently in cells that have already undertaken their differentiation program and suggesting the possibility that the up-modulation of p63 by 16E5 could be a consequence of KGFR down-regulation. Consistent with this possibility, the receptor overexpression in KGFR cells was able to induce a decrease of p63 independently from TG stimulus (Fig. 6c). Thus, KGFR is involved in the balance of p63 expression and this crosstalk may be responsible for the impairment of keratinocyte differentiation induced by 16E5.

To evaluate the role of receptor activation and signaling on p63 modulation, HaCaT E5 and HaCaT KGFR/E5 cells were treated with TG, serum starved and stimulated with KGF as above. Quantitative real-time RT-PCR showed a ligand-dependent decrease of p63 mRNA in KGFR/E5 (Fig. 6d); in contrast, the up-modulation of p63 transcript in E5 cells was not affected by KGF stimulation, consistent with the ability of 16E5 to induce KGFR down-modulation (Fig. 6d). Again, the decrease of p63 expression in KGFR/E5 cells compared to cells expressing 16E5 alone (Fig. 6d) can be explained by the partial persistence of the receptor signaling upon starvation. The ability of KGFR to specifically counteract the up-modulating effect of 16E5 on p63 was also validated at the protein level by Western blot analysis: the band corresponding to p63 appeared decreased in a ligand-dependent manner in KGFR/E5, but not in E5 cells (Fig. 6e).

Since the PI3K/Akt pathway is crucial for the KGFR role in 16E5 expressing cells, we wondered if this signaling pathway is also involved in the KGFR-induced down-regulation of p63. To address this point, HaCaT E5 and HaCaT KGFR/E5 cells were serum starved, treated with TG and then stimulated with KGF in presence or not of the Akt inhibitor as reported in Materials and Methods. Quantitative real-time RT-PCR clearly showed that Akt inhibition increased the p63 expression in KGFR/E5 cells, blocking the receptor effect, while it did not interfere with the p63 up-modulation induced by the viral protein in E5 cells (Fig. 6f), suggesting that the PI3K/Akt pathway plays a role in the KGFR-mediated down-modulation of p63.

### KGFR down-regulates p63 through a miR-203 independent mechanism

It has been demonstrated that, during differentiation, p63 is specifically down-regulated by miR-203 ^28, 29^. Since it has been recently reported that 16E5 expression in keratinocytes up-regulates p63 through down-regulation of miR-203 [26], we wondered whether the decrease of p63 observed upon KGFR expression in our cellular model could be a consequence of an opposite modulation exerted by the viral protein and the receptor on miR-203. Quantitative real-time RT-PCR in HaCaT pCI-neo, HaCaT E5 and HaCaT KGFR cells confirmed the down-modulation of miR-203 in cells expressing 16E5 and indicated that this modulation was evident upon differentiation following TG-treatment (Fig. 6g). These results are consistent with the p63 up-regulation induced by 16E5 in differentiating cells (Fig. 6c). However, in HaCaT KGFR cells, miR-203 was not up-modulated, but decreased if compared to control cells (Fig. 6g), indicating that the receptor-mediated down-modulation of p63 (Fig. 6c) is regulated by a miR-203 independent mechanism.

Finally, the analysis of miR-203 expression level was assessed in KGFR/E5 doubly transfected cells and compared to that observed in E5 singly transfected. The results confirmed that KGFR overexpression was not able to contrast the 16E5-mediated down-modulation of miR-203 (Fig. 6h), further indicating that the effect of KGFR on p63 (Fig. 6c-f) is not controlled by miR-203.

## DISCUSSION

The possibility that HPV16 viral protein E5 and the receptor tyrosine kinase KGFR/FGFR2b might be inversely correlated in their expression, exerting opposite and interplaying roles in epithelial homeostasis and tumorigenesis, was the starting hypothesis of this study. In our previous paper, aimed to address the role played by the two proteins in epithelial growth, we have already reported that, in 16E5 expressing keratinocytes, transcriptional down-modulation of KGFR led to a reduction of the ligand-dependent proliferation, suggesting a functional crosstalk [5]. However, because KGFR and its ligands, acting mostly on confluent and suprabasal cells, are key mediators of the physiological epithelial differentiation [6, 10, 30, 31] and because also the perturbing function of 16E5 on cell growth and stratification [23] is believed to occur on differentiating cells [3, 4], here we focused on the molecular mechanisms which may control the KGFR/16E5 interplay, using an in vitro model developed to monitor the contributions of their gene expression and activated pathways in keratinocytes committed to differentiate. In particular, the treatment of E5-transfected HaCaT cells with TG, which is able to generate an homogenous population of differentiating cells expressing increasing amount of KGFR and K1 [10], allowed us to obtain a cell model system close to that achieved expressing E5 in organotypic HaCaT raft cultures [23]: in fact our results, showing the dose-dependent decrease of K1 expression induced by 16E5, are in agreement with the decrease of K1 expression observed in the suprabasal layer of the organotypic cultures [23]. However, we were also able to correlate the down-regulation of K1 transcripts with a parallel down-modulation of KGFR mRNA and protein, which was much more evident in cells committed to differentiation upon TG-treament compared to untreated undifferentiated cultures, suggesting that this KGFR down-modulation would be the molecular event responsible for the 16E5-mediated decrease of differentiation.

Since it is possible to control keratinocyte early differentiation through the forced expression or depletion of KGFR 10, we used here the same experimental strategy to evaluate the contribution of receptor expression and signaling on the 16E5 effects, demonstrating that the synchronous up- or down-modulation of KGFR induces a corresponding modulation of K1 expression in the presence of E5 and under a wave of differentiation. Interestingly, we found that the induction of KGFR expression and the triggering of receptor kinase activation and signaling are capable to efficiently counteract the 16E5 impairment of early differentiation. These observations are in agreement with the results obtained in models of epithelial tumor growth in nude mice, in which re-expression of KGFR in the cancer cells led to a reduction of their proliferation and enhancement of differentiation [11, 12]. Therefore, our present results provide a further demonstration of the tumor-suppressive role of KGFR in the new unexplored context of carcinogenesis related to the activity of the HPV16 E5 oncogene.

One of the main molecular mechanisms known to drive the shift from the basal to suprabasal layers of stratified epithelial tissues is the down-modulation of the transcription factor p63, and in particular of its isoform ΔNp63, target of the miR-203 [27]. The HPVs, which require for their replication the maintenance of proliferation in differentiating cells, appear to control p63 expression through either E7 or E5 oncoproteins: to do this, both proteins down-regulate the cellular miR-203 [26, 32]. In agreement with these studies, our findings confirmed that also in our cell model E5 enhances the p63 expression down-regulating miR-203. In addition, the results demonstrate that the viral protein activity is more efficient in TG-treated than in untreated cells, providing evidence of the modulation of genes such as KGFR and p63 in cells already committed to differentiation. On the other hand, we found that also KGFR is involved in the balance of p63, inducing a ligand-dependent decrease of p63 transcription, which is regulated by a miR-203 independent mechanism.

Among the possible pathways activated by the receptor, which may play a role in the perturbation of the epithelial homeostasis induced by E5, we focused on the PI3K/Akt signaling, because it seems to be the main pathway implicated in the control of KGFR-dependent keratinocyte differentiation [10]: the results demonstrate that the shut-down of KGFR-mediated PI3K/Akt signaling is a crucial step for 16E5-mediated impairment of early differentiation and that this may occur as a consequence of the receptor down-modulation. Moreover, we found that Akt inhibition blocked the modulating effect on p63 expression induced by the receptor, but not by E5, suggesting that KGFR down-regulates p63 and up-regulates K1 through the PI3K/Akt pathway.

The existence of a KGFR/p63 crosstalk in either direction is not surprising in light of several studies which have reported that p63, and in particular its isoform ΔNp63, may directly regulate KGFR/FGFR2b transcription [33-35]. Alteration of this crosstalk may be responsible for the impairment of keratinocyte differentiation induced by E5 and leads to the hypothesis that the tumor-suppressive action of KGFR and the opposite oncogenic function of E5 might both involve p63. In this scenario, E5 might be able to up-modulate p63 through two independent distict pathways: the first, well established, mediated by miR-203 and the second, miR-203 independent, triggered by down-modulation of KGFR expression and signaling.

## MATERIALS AND METHODS

### Cells and treatments

The human keratinocyte cell line HaCaT [17] was cultured in Dulbecco's DMEM, supplemented with 10% fetal bovine serum (FBS) plus antibiotics. The subclone W12p6 of the human cervical keratinocyte cell line W12 initiated from a low-grade cervical lesion [18], which retain 100 to 200 copies of the HPV16 episomes per cell [18-20], was cultured as previously described [18]. HaCaT cells were transiently transfected or cotransfected with pCI-neo expression vector containing 16E5-HA [21] (HaCaT E5), human KGFRwt (HaCaT KGFR WT) or a kinase negative mutant KGFRY656F/Y657F (HaCaT KGFRkin^−^) [22] using jetPEI^TM^ DNA Trasfection Reagent (Polyplus-trasfection, New York, NY, USA) according to manufacturer's instructions.

For simultaneous 16E5 expression and KGFR silencing, HaCaT cells were cotransfected with pCI-neo 16E5-HA and Bek small interfering RNA (KGFR siRNA) (Santa Cruz Biotechnology Inc., Santa Cruz, CA, USA), or with 16E5-HA and unrelated siRNA as a control, using Lipofectamine 2000 Transfection Reagent (Invitrogen, Carlsbad, CA, USA) according to the manufacturer's protocol.

For growth factor stimulation, cells were serum starved and then incubated with 20 ng/ml KGF (Upstate Biotechnology, Lake Placid, NY, USA) for 24h at 37°C.

To inhibit Akt, cells were incubated with the specific Akt inhibitor 1L-6-hydroxy-methyl-chiro-inositol 2-(R)-2-O-methyl-3-O-octadecylcarbonate (Calbiochem, San Diego, CA, USA) 1 μM for 1 h at 37°C before treatment with KGF in the presence of the inhibitor.

To induce the differentiation program in pre-confluent conditions, HaCaT cells were incubated with different doses of Thapsigargin (TG) (0,1 μM, 0.5 μM, 1 μM) (Molecular Probes, Eugene, OR, USA) for 1 h at 37°C, followed by incubation at 37°C for 36 h. Since TG stock (1 mg/ml) was diluted in solvent dimethyl sulfoxide (DMSO), control cells were treated with an equal amount of DMSO.

### Microinjection

Microinjection was performed with an Eppendorf microinjector (Eppendorf, Hamburg, Germany) and an inverted microscope (Zeiss, Oberkochen, Germany). A mixture of 100 nM Bek siRNA (Santa Cruz Biotechnology), 100 ng/ml pCI-neo 16E5-HA and 1 mg/ml mouse IgG (Cappel Research Products, Durham, NC, USA) in distillate water were microinjected in the cytoplasm to simultaneously induce RNA interference and consequent KGFR silencing and 16E5 overexpression. Unrelated siRNA was microinjected as negative control.

### Immunofluorescence

Cells, grown on coverslips, were fixed with 4% paraformaldehyde in PBS for 30 minutes at 25°C and permeabilized as described [5]. Cells were then incubated for 1h at 25°C with the following primary antibodies: mouse monoclonal anti-HA (1:50 in PBS; Covance, Berkeley, CA, USA) and rabbit polyclonal anti-K1 (1:50 in PBS; Covance). The primary antibodies were visualized using goat anti-mouse IgG-FITC (1:20 in PBS; Cappel) and goat anti-rabbit IgG-Texas Red (1:200 in PBS; Jackson Immunoresearch Laboratories, West Grove, PA, USA) for 30 minutes at 25°C. Nuclei were stained with DAPI (1:1000 in PBS; Sigma-Aldrich Inc., Saint Louis, MO, USA). Fluorescence signals were analyzed by scanning cells in sequential sections with an ApoTome System (Zeiss); image analysis was performed by the Axiovision software (Zeiss) and 3D reconstruction of a selection of three central optical sections was shown in each figure. The fluorescence intensity of the signals was performed by the analysis of 50 cells for each sample in five different microscopic fields from three different experiments and the cut-off of the signal intensity was selected for both TG-treated and control samples in order to discriminate between K1 positive and negative cells using the KS300 3.0 Image Processing System (Zeiss). Quantitative analysis was assessed counting for each sample a total of 50 cells, randomly observed in 10 microscopic fields from three different experiments. Results have been expressed as mean values ± standard errors (SE); p values were calculated using Student's t test and significance level has been defined as p<0.05

### Western blot analysis

HaCaT cells were lysed as described [10]; 50 μg of total protein were resolved under reducing conditions by 8% SDS-PAGE and transferred to reinforced nitrocellulose (BA-S 83, Schleider and Schuell, Keene, NH, USA). The membranes were blocked with 5% non fat dry milk in PBS 0.1% Tween 20, and incubated with anti-Bek (C-17, Santa Cruz) polyclonal antibodies, anti-K1 (Covance) polyclonal antibodies and p63 monoclonal antibody (4A4, Santa Cruz). The membranes were rehydrated and probed again with anti-actin (Sigma) monoclonal antibody, to estimate the protein equal loading. Densitometric analysis was performed using Quantity One Program (Bio-Rad Laboratoires, Hercules, CA, USA). The resulting values from three different experiments were then normalized and expressed as fold increase respect to the control value.

### Primers

Oligonucleotide primers for target genes and for the housekeeping gene were chosen with the assistance of the Oligo 5.0 computer program (National Biosciences, Plymouth, MN, USA) and purchased from Invitrogen. The following primers were used: for *FGFR2b/KGFR* target gene: 5'-CAGGGGTCTCCGAGTATGAA-3 (sense), 5'-TCTAAAGGCAACCTCCGAGA-3' (anti-sense); for *HPV 16E5* gene 5'-CGCTGCTTTTGTCTGTGTCT-3' (sense), 5'-GCGTGCATGTGTATGTATTAAAAA-3' (antisense); for *K1* target gene 5'-AGCACAAGCCACACCACCATC-3' (sense), 5'-CGCCACCTCCAGAACCATAGC-3' (antisense); for *p63* target gene 5'- CGCCGCAATAAGCAACAG -3' (sense), 5'- GTAGCCTCTTACTTCTCCTTCC-3' (antisense) (designed to recognize both ΔNp63 and TAp63 α and β isoforms); for the β-actin housekeeping gene: 5'-CATCAGCAATGCCTCCTGCAC-3' (sense), 5'-GTCATGAGTCCTTCCACGATACCAA-3' (antisense). For each primer pair, we performed no-template control and no-reverse-transcriptase control (RT negative) assays, which produced negligible signals. For microRNA Taqman assays, primers and probes were provided by Applied Biosystems (Applied Biosystems; Foster City, CA, USA).

### RNA extraction and cDNA synthesis

RNA was extracted using the TRIzol method (Invitrogen) according to manufacturer's instructions and eluted with 0,1% diethylpyrocarbonate (DEPC)-treated water. Each sample was treated with DNAase I (Invitrogen). Total RNA concentration was quantitated by spectrophotometry. 1 μg of total RNA was used to reverse transcription using iScript^TM^ cDNA synthesis kit (Bio-Rad) according to manufacturer's instructions. For microRNA Taqman assays, 2.5 ng of total RNA were reverse transcribed using Taqman^®^ MicroRNA Reverse Transcription Kit (Applied Biosystems).

### PCR amplification and real-time quantitation

Real-time PCR was performed using the iCycler Real-Time Detection System (iQ5 Bio-Rad) with optimized PCR conditions. The reaction was carried out in 96-well plate using iQ SYBR Green Supermix (Bio-Rad) adding forward and reverse primers for each gene and 1 μl of diluted template cDNA to a final reaction volume of 15 μl. All assays included a negative control and were replicated three times. The thermal cycling programme was performed as described ^5^. Real-time quantitation was performed with the help of the iCycler IQ optical system software version 3.0a (Bio-Rad), according to the manufacturer's manual. For microRNA Taqman assays, relative quantities of mature microRNAs were determined using Applied Biosystems TaqMan microRNA Assays (Applied Biosystems). Results are reported as mean ± standard deviation (SD) from three different experiments in triplicate.
